# Numerical Investigations through ANNs for Solving COVID-19 Model

**DOI:** 10.3390/ijerph182212192

**Published:** 2021-11-20

**Authors:** Muhammad Umar, Zulqurnain Sabir, Muhammad Asif Zahoor Raja, Shumaila Javeed, Hijaz Ahmad, Sayed K. Elagen, Ahmed Khames

**Affiliations:** 1 Department of Mathematics and Statistics, Hazara University, Mansehra 21300, Pakistan; umar_maths@hu.edu.pk; 2 Future Technology Research Center, National Yunlin University of Science and Technology, 123 University Road, Section 3, Douliou 64002, Taiwan; rajamaz@yuntech.edu.tw; 3 Department of Mathematics, Islamabad Campus, COMSATS University Islamabad, Park Road, Islamabad 45550, Pakistan; 4 Department of Computer Engineering, Biruni University, Istanbul 34025, Turkey; hijaz555@gmail.com; 5 Section of Mathematics, International Telematic University Uninettuno, Corso Vittorio Emanuele II, 39, 00186 Roma, Italy; 6 Department of Mathematics and Statistics, College of Science, Taif University, P.O. Box 11099, Taif 21944, Saudi Arabia; skhalil@tu.edu.sa; 7 Department of Pharmaceutics and Industrial Pharmacy, College of Pharmacy, Taif University, P.O. Box 11099, Taif 21944, Saudi Arabia; A.khamies@tu.edu.sa

**Keywords:** COVID-19 spreading model, artificial neural networks, Levenberg-Marquardt backpropagation, reference dataset, numerical results

## Abstract

The current investigations of the COVID-19 spreading model are presented through the artificial neuron networks (ANNs) with training of the Levenberg-Marquardt backpropagation (LMB), i.e., ANNs-LMB. The ANNs-LMB scheme is used in different variations of the sample data for training, validation, and testing with 80%, 10%, and 10%, respectively. The approximate numerical solutions of the COVID-19 spreading model have been calculated using the ANNs-LMB and compared viably using the reference dataset based on the Runge-Kutta scheme. The obtained performance of the solution dynamics of the COVID-19 spreading model are presented based on the ANNs-LMB to minimize the values of fitness on mean square error (M.S.E), along with error histograms, regression, and correlation analysis.

## 1. Introduction

The development of science, both over technology, as well as information, is related to the human’s heathy life. In fact, there are numerous complications that are not considered easy in an individual’s life. In the health sector, a number of infectious diseases are produced by the bacterial viruses. The diseased infections are a big danger for the society that always disturbed the economies of the countries, destroyed the sector of education and demolished the tourism industry. A novel Coronavirus is a dangerous transmittable virus discovered at the end of the 19th century and spread all around the world [1]. Many peoples died from coronavirus disease (COVID-19) and the positive cases, along with the recovery rate, has also a great number [1]. The common COVID-19 symptoms are runny noses, sore throats, coughs, headaches and fever, or severe breathing symptoms, such as bleeding, high fever, cough with phlegm, shortness of breath, and chest pain [2].

In recent decades, the researchers considered a favorite topic examining the dynamics of coronavirus, and they presented many recommendations. Donders et al. [3] expressed the International Society of Infectious Disease in Obstetrics and Gynecology (ISIDOG) based recommendations for the COVID-19. Wang [4] suggested a mathematical COVID-19 system based on the limitations, applications, and potentials. Rhodes et al. [5] designed a mathematical system to express the public difficulties of the infection control of coronavirus. Javeed et al. [6] presented a novel model for COVID-19, including the effects of government strategies and comparative analysis of different countries regarding prevention of disease. Jewell et al. [7] described the potential impacts of disruption-based human immunodeficiency virus (HIV) programs in Africa produced by the coronavirus. Sánchez et al. [8] suggested a fractal susceptible, infection, treatment, and recovery (SITR) nonlinear model to express the coronavirus dynamics. Khrapov et al. [9] comparatively analyzed the mathematical system using the dynamics of the coronavirus epidemic growth in various countries. Elsonbaty et al. [10] proposed a dynamical discrete SITRs fractional system related to coronavirus. Thompson [11] introduced the epidemiologic system that is considered a significant tool based on the coronavirus interferences. Umer et al. [12,13] calculated the numerical results based on the swarming and heuristic schemes for solving the nonlinear SITR model based on the coronavirus.

Kharis et al. [14] studied a mathematical model, which plays a vital role to avoid the spread of viruses. Yulida [15] explained that a significant role of mathematics is observed to explore the outbreak of the dynamics of diseases, spreading and forecasting patterns to deal stratagems called as epidemiological mathematics. Moreover, the solutions of some mathematical models are presented analytically that involves epidemic diseases. Hence, it is important to gain the numerical designs for such problems. Therefore, the stochastic computational schemes using the artificial neuron networks (ANNs) with the novel features of the Levenberg-Marquardt backpropagation (LMB), i.e., ANNs-LMB, is implemented to solve the nonlinear COVID-19 spreading model. The stochastic procedures of ANNs-LMB have never been applied for solving the nonlinear COVID-19 spreading model. The data ratio is adjusted for three cases of the coronavirus spreading model are 80%, 10%, and 10% for training, testing and validation, respectively. The numerical measures are performed using the ANNs-LMB for solving the coronavirus spreading model and comparison will be performed through the reference dataset based Runge-Kutta scheme [16]. Some reputed used of stochastic procedures are the delay differential models [16,17], multi-fractional systems [18,19,20], prey-predator model [21], infection based HIV system [22], singular functional systems [23], Thomas-Fermi equation [24], heat conduction model [25], mosquito dispersal system [26], and periodic singular model [27,28].

The aim of the study is to present a design of computing framework based on artificial neural network trained with Livenberg-Marquardt backpropagation (ANNs-LMB) for analysis of coronavirus spreading model. While a lot of literature is available on the internet used for reliable and accurate prediction of coronavirus spreading in different regions of the world using sophisticated computing paradigm of neural networks, deep-learning, and transfer learning, combined with deterministic and stochastic optimization solver for global and local search, including a novel hybrid time series model of COVID-19 [29], neural network prediction of COVID-19 pandemic at the Brazilian Amazon [30], integrated neuro-evolution heuristics approach [31], transfer learning based computing [12], deep neural networks [32], nonlinear autoregressive networks [33], and radial base networks [34]. However, the potentials of the proposed ANNs-LMB to solve the coronavirus spreading model are presented as follows for better understanding of the contribution:

- Artificial intelligence (AI) knacks-based computational procedure via neural networks models learned with Livenberg-Marquardt algorithm is introduced and implemented to solve nonlinear coronavirus spreading model represented with 7 classes based systems of ordinary differential equations (ODEs).
- The comparison of the results obtained through designed computing ANNs-LMB with numerical solutions are found in good agreement on the basis of absolute error (AE) values, which approve the value, worth and significance of the ANNs-LMB to solve the nonlinear coronavirus spreading model.
- The performance or convergence curves on mean square error (MSE), regression metric calculations of correlation index, and error histograms (EHs) through exhaustive simulations further indorse the reliability and consistency of the ANNs-LMB scheme.

The remaining parts are organized as: The designed methodology based ANNs-LMB is provided in Section 2, numerical simulations are presented in Section 3, and concluding remarks are provided in Section 4.

## 2. Material and Methods

The material and methodologies presented here are described in three portions for solving the nonlinear coronavirus spreading model as follows.

The nonlinear COVID-19 spreading model has seven classes, susceptible (*S*(*y*)), exposed population (*E*(*y*)), infected (*I*(*y*)), removed (*R*(*y*)), total population (*N*(*y*)), public perception (*D*(*y*)), and cumulative case (*C*(*y*)), along with the initial conditions (ICs), given as [28]:

(1) $$\left\{ \begin{array}{ll} S^{\prime }(y)=-\frac{\beta_{0}E(y)S(y)}{N(y)}-\frac{\beta (y)I(y)S(y)}{N(y)}-\mu S(y), & S(0)=I_{1},\\ E^{\prime }(y)=\frac{\beta_{0}E(y)S(y)}{N(y)}+\frac{\beta (y)I(y)S(y)}{N(y)}-(\mu +\sigma )E(y), & E(0)=I_{2},\\ I^{\prime }(y)=\sigma E(y)-(\mu +\gamma )I(y), & I(0)=I_{3},\\ R^{\prime }(y)=-\mu R(y)+\gamma I(y), & R(0)=I_{4},\\ N^{\prime }(y)=-\mu N(y), & N(0)=I_{5},\\ D^{\prime }(y)=-\lambda D(y)+d\gamma I(y), & D(0)=I_{6},\\ C^{\prime }=\sigma E(y), & C(0)=I_{7}. \end{array}\right.$$

where μ, $\beta_{0}$, and σ are the emigration, initial transmission, and latent rates, whereas the transmission rates at time *y*, public reaction, infected, and severe cases are β(y), λ, γ, and *d*. The ICs are *I*_1_, *I*_2_, *I*_3_, *I*_4_, *I*_5_, *I*_6_, and *I*_7_.

In the first step, the necessary details of the numerical procedure of the Runge-Kutta solver are provided which are used to create dataset for the system (1). In the second part, designed ANNs-LMB computing platform in terms of networks modeling, layer structure, and backpropagation algorithm are provided, while, in the third step, implementation procedures of the proposed ANNs-LMB to solve the nonlinear coronavirus spreading model is provided.

The reference datasets for different scenario of nonlinear coronavirus spreading model represented with 7 class-based systems of ODEs as portrayed in the set of equations in Equation (1) are calculated by exploiting the numerical strength of Adams solver using ‘NDSolve’ routine for solution of differential equations in Mathematica software package in Microsoft Windows 10 environment. The default parameter settings of Adams procedure, i.e., good accuracy, tolerances, stoppage criteria, etc., is used for execution. The dataset generated is used for modeling the networks with different distributions of data in training, testing, and validation samples for each case of the nonlinear corona virus spreading system (1).

**Figure 1:**
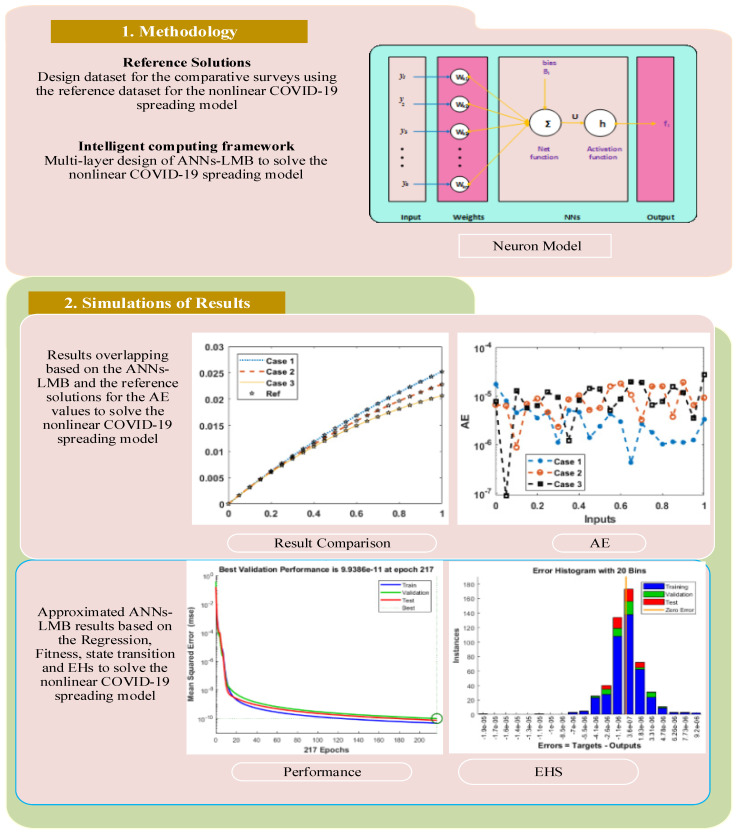
Workflow diagram using the ANNs-LMB to solve the nonlinear COVID-19 spreading model.

The appropriate work flow structure of proposed methodology ANNs-LMB in terms of problem, layer structure, and results description with performance analysis, and optimization procedures are illustrated in Figure 1.

**Figure 2:**
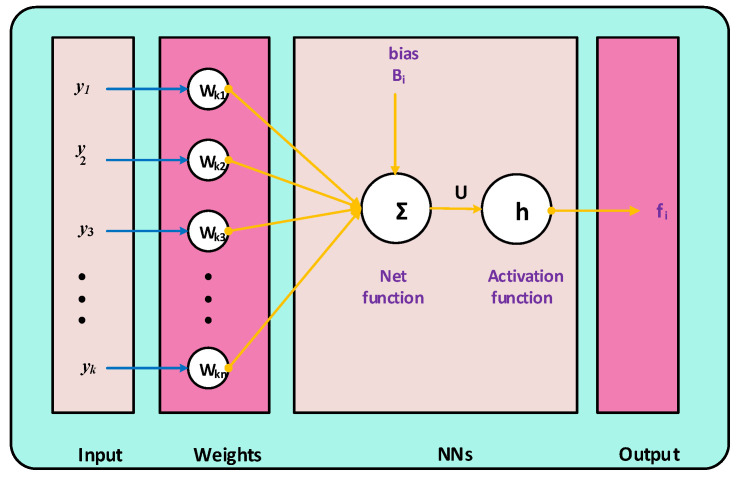
Structure of a single neuron based on the ANNs-LMB.

The proposed model for a single neuron representation is shown in Figure 2. The build-in ‘nftool’ command available in the neural network toolbox in MATLAB software package in Microsoft Windows 10 environment is used for the training, testing, and validation with 80%, 10%, and 10% samples, respectively, for formulation of networks to find the approximate solutions for nonlinear coronavirus spreading model represented for the 7 class-based systems of ordinary differential equations as given in set (1). Moreover, the number of epochs executed for each problem is set after detailed simulations, as per the procedure provided in references [35,36].

## 3. Numerical Experimentation with Interpretation of Results

This section presents the numerical performances of three cases based on the nonlinear coronavirus spreading model using the proposed ANNs-LMB. The mathematical form of each case is given as:

**Case 1.** Considering the nonlinear coronavirus spreading model with the appropriate values is shown as:
  (2) $$\left\{ \begin{array}{ll} S^{\prime }(y)=-\frac{0.5E(y)S(y)}{N(y)}-\frac{0.6I(y)S(y)}{N(y)}-0.0205S(y), & S(0)=0.9,\\ E^{\prime }(y)=\frac{0.5E(y)S(y)}{N(y)}+\frac{0.6(y)I(y)S(y)}{N(y)}-(0.0205+\frac{1}{3})E(y), & E(0)=0.1,\\ I^{\prime }(y)=\frac{1}{3}E(y)-(0.0205+\frac{1}{5})I(y), & I(0)=0,\\ R^{\prime }(y)=-0.0205R(y)+\frac{1}{5}I(y), & R(0)=0,\\ N^{\prime }(y)=-0.0205N(y), & N(0)=14,\\ D^{\prime }(y)=-\frac{1}{11.2}D(y)+0.04\gamma I(y), & D(0)=0,\\ C^{\prime }(y)=\frac{1}{3}E(y), & C(0)=0. \end{array}\right.$$
**Case 2.** Considering the nonlinear coronavirus spreading model with the appropriate values is shown as:
  (3) $$\left\{ \begin{array}{ll} S^{\prime }(y)=-\frac{0.5E(y)S(y)}{N(y)}-\frac{0.6I(y)S(y)}{N(y)}-0.1205S(y), & S(0)=0.9,\\ E^{\prime }(y)=\frac{0.5E(y)S(y)}{N(y)}+\frac{0.6(y)I(y)S(y)}{N(y)}-(0.1205+\frac{1}{3})E(y), & E(0)=0.1,\\ I^{\prime }(y)=\frac{1}{3}E(y)-(0.1205+\frac{1}{5})I(y), & I(0)=0,\\ R^{\prime }(y)=-0.1205R(y)+\frac{1}{5}I(y), & R(0)=0,\\ N^{\prime }(y)=-0.1205N(y), & N(0)=14,\\ D^{\prime }(y)=-\frac{1}{11.2}D(y)+0.04\gamma I(y), & D(0)=0,\\ C^{\prime }(y)=\frac{1}{3}E(y), & C(0)=0. \end{array}\right.$$
**Case 3.** Considering the nonlinear coronavirus spreading model with the appropriate values is shown as:
  (4) $$\left\{ \begin{array}{ll} S^{\prime }(y)=-\frac{0.5E(y)S(y)}{N(y)}-\frac{0.6I(y)S(y)}{N(y)}-0.2205S(y), & S(0)=0.9,\\ E^{\prime }(y)=\frac{0.5E(y)S(y)}{N(y)}+\frac{0.6(y)I(y)S(y)}{N(y)}-(0.2205+\frac{1}{3})E(y), & E(0)=0.1,\\ I^{\prime }(y)=\frac{1}{3}E(y)-(0.2205+\frac{1}{5})I(y), & I(0)=0,\\ R^{\prime }(y)=-0.2205R(y)+\frac{1}{5}I(y), & R(0)=0,\\ N^{\prime }(y)=-0.2205N(y), & N(0)=14,\\ D^{\prime }(y)=-\frac{1}{11.2}D(y)+0.04\gamma I(y), & D(0)=0,\\ C^{\prime }(y)=\frac{1}{3}E(y), & C(0)=0. \end{array}\right.$$

**Figure 3:**
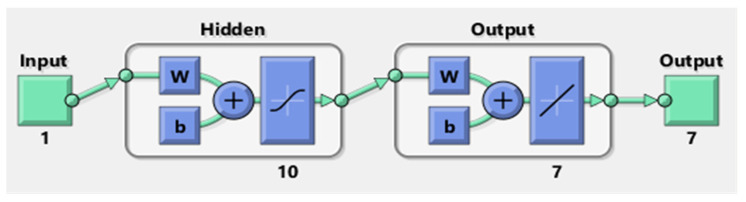
Proposed ANNs-LMB to solve the nonlinear COVID-19 spreading model with a single input layer, a single hidden layer with 10 number of neurons, and a single output layer with 7 outputs.

The numerical values to solve each class of the nonlinear coronavirus spreading model are provided through the procedures of ANNs-LMB with input interval [0,1] and 0.01 step size. The designed ANNs-LMB procedures is selected as a larger part, i.e., for (training—80%), (validation—10%), and (testing—10%), respectively. The hidden neurons have been selected as 10 for each case of the nonlinear coronavirus spreading model [37], and the obtained numerical performances through the ANNs-LMB to solve each case of the model are presented in Figure 3 based on the single layer structure [38].

**Figure 4:**
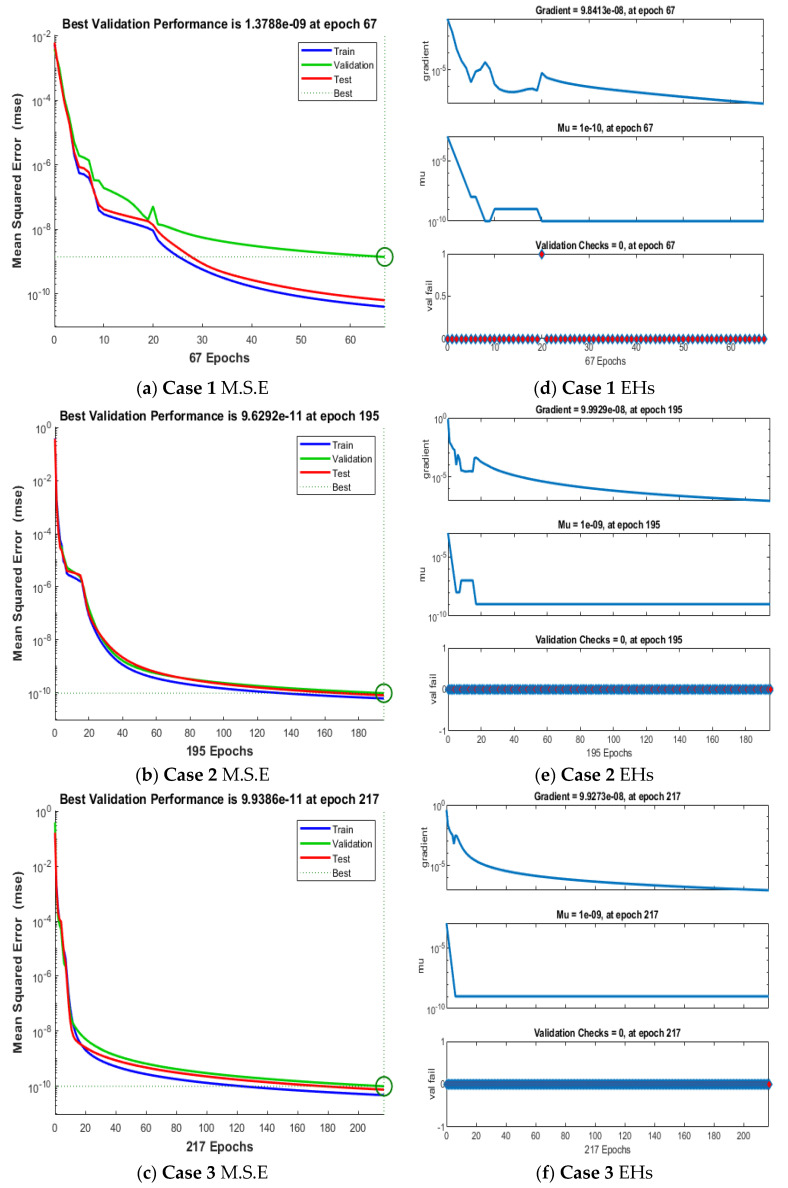
Performances of M.S.E (**a**–**c**) and State transition (**d**–**f**) to solve the nonlinear COVID-19 spreading model.

**Figure 5:**
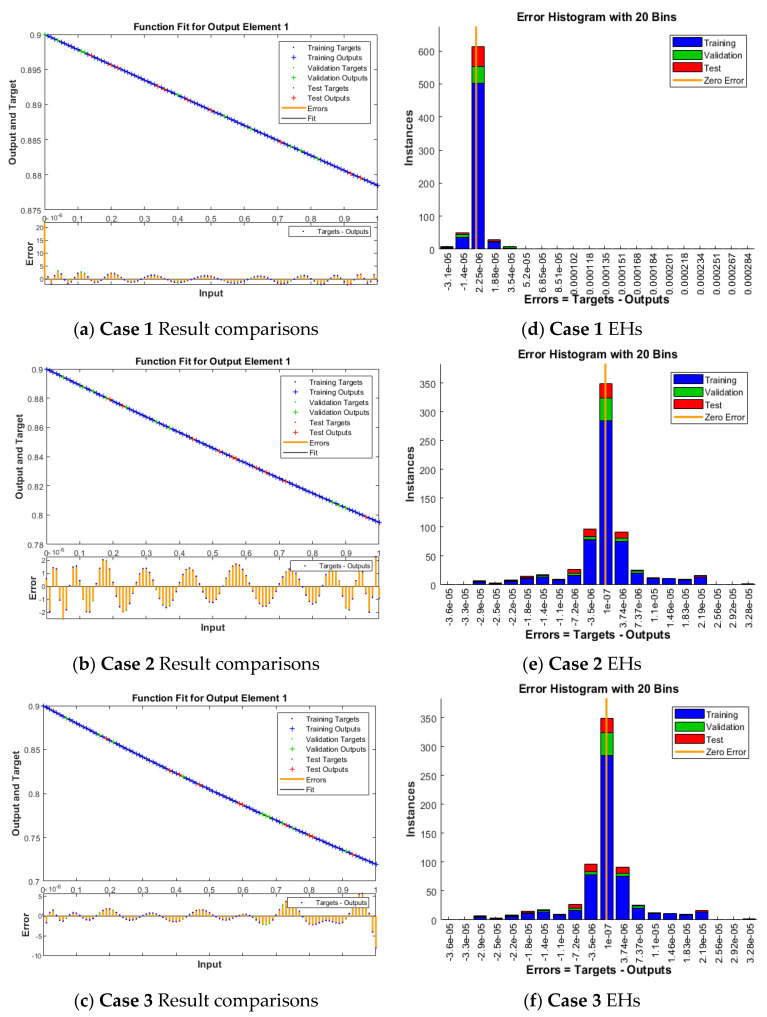
Results comparison and EHs to solve the nonlinear COVID-19 spreading model.

**Figure 6:**
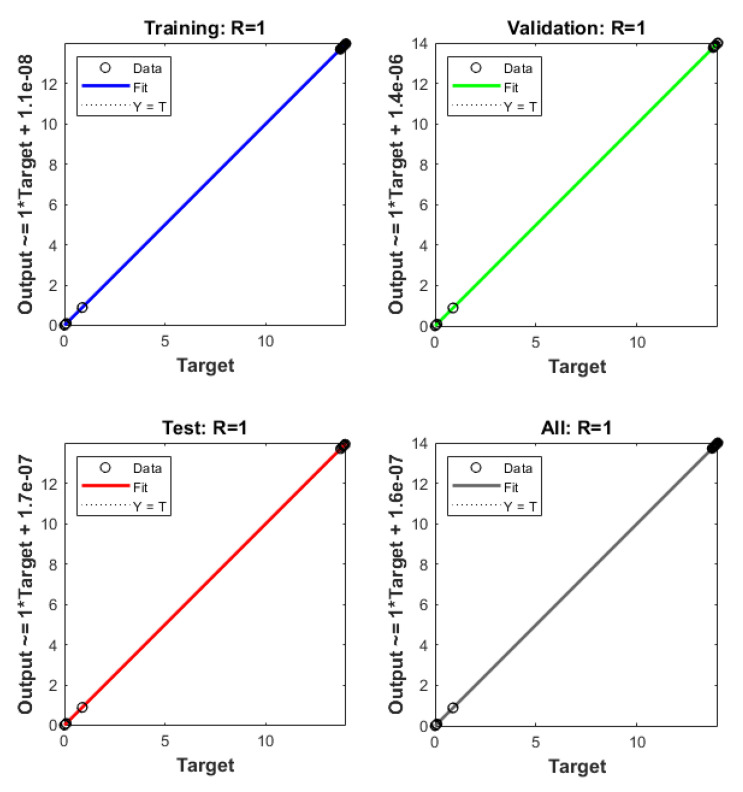
**Case 1** Regression plots based on the nonlinear COVID-19 spreading model.

**Figure 7:**
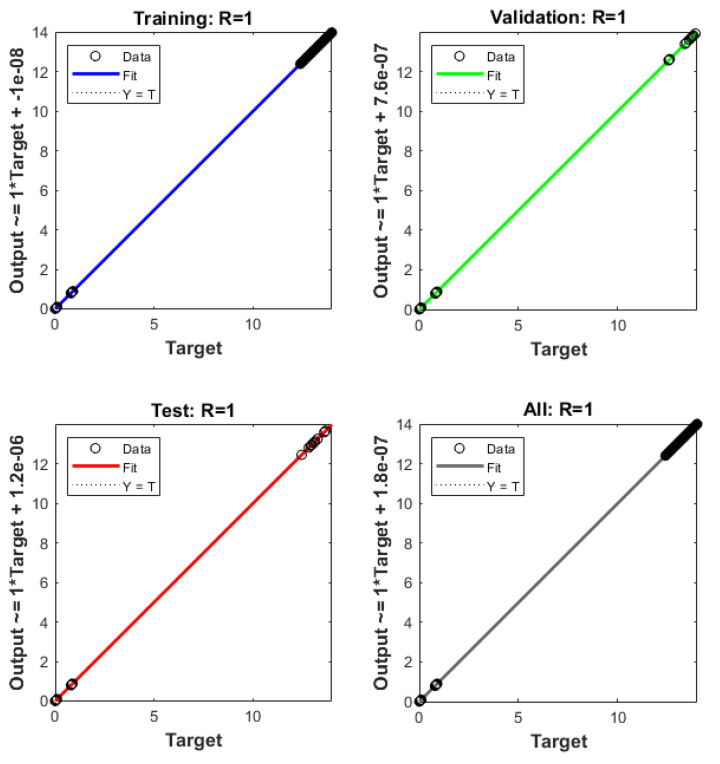
**Case 2** Regression plots based on the nonlinear COVID-19 spreading model.

**Figure 8:**
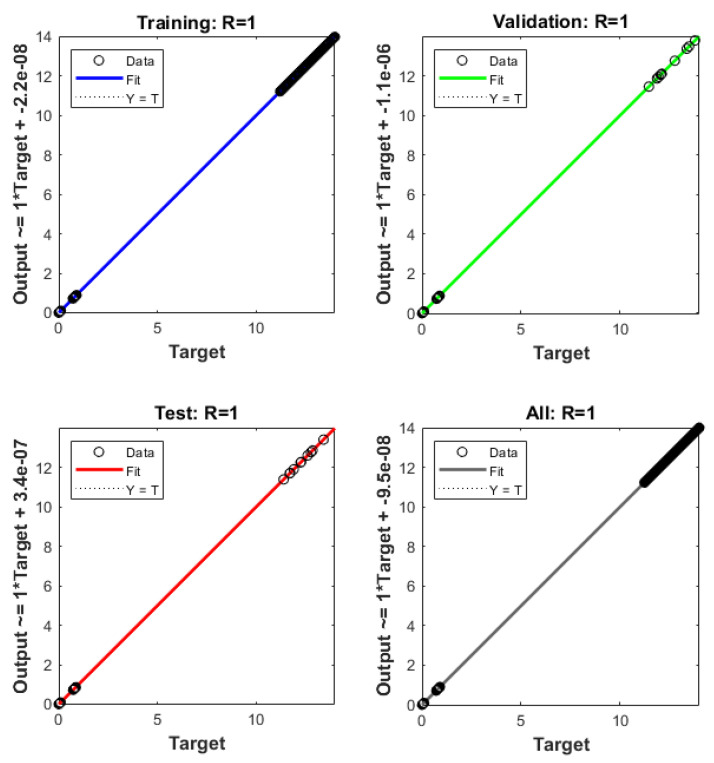
**Case 3** Regression plots based on the nonlinear COVID-19 spreading model.

The plots of the ANNs-LMB to solve the nonlinear coronavirus spreading model are drawn in Figure 4, Figure 5, Figure 6 and Figure 7. The proficient performances, as well as states of transition, to solve each class of the nonlinear coronavirus spreading model is drawn in Figure 4. The obtained numerical performances are plotted based on the M.S.E for testing, best curves, validation, and training in Figure 4a–c to solve the nonlinear coronavirus spreading model. These illustrations show that ANNs-LMB to solve each nonlinear coronavirus spreading model at epochs numbers 67, 195, and 217 with MSE almost 1.37 × 10^−9^, 9.62 × 10^−11^ and 9.93 × 10^−11^, respectively. Figure 4d–f indicates the gradient performances using the ANNs-LMB to solve the nonlinear coronavirus spreading model which found around 9.98 × 10^−8^, 9.99 × 10^−8^, and 9.92 × 10^−8^ for the respective three cases. These graphs show the precision, convergence, and accuracy of the ANNs-LMB to solve each variant of the nonlinear coronavirus spreading model. The plots based on the fitting curves to solve each variant of the nonlinear coronavirus spreading model are drawn in Figure 5a–c, which show the accuracy through the comparative ANNs-LMB results with the reference data. The error plots are demonstrated through the measures of training, testing, and verification and using the ANNs-LMB to solve each variant of the nonlinear coronavirus spreading model. The EHs plots are provided in Figure 5d–f, which show that the errors lie around −2.2 × 10^−6^, 1.0 × 10^−7^, and 1.01 × 10^−7^ for the three respective cases of coronavirus spread model. The regression illustrations are provided in Figure 6, Figure 7 and Figure 8 based on each variant of the nonlinear coronavirus spreading model. These correlation plots are presented with the regression index value close to unity in each case. The correlation values are observed nearly equal to 1 for each case of the nonlinear coronavirus spreading model which demonstrate near to perfect modeling of the solution dynamics of the system. The testing, training, and authentication plots indicate the accuracy and precision of the ANNs-LMB to solve each class of the nonlinear coronavirus spreading model. Furthermore, the convergence through the MSE procedures is sanctioned through the epochs, training, verification, backpropagation presentations, complexity, and testing measures are drawn in Table 1 to solve the nonlinear coronavirus spreading model.

**Figure 9:**
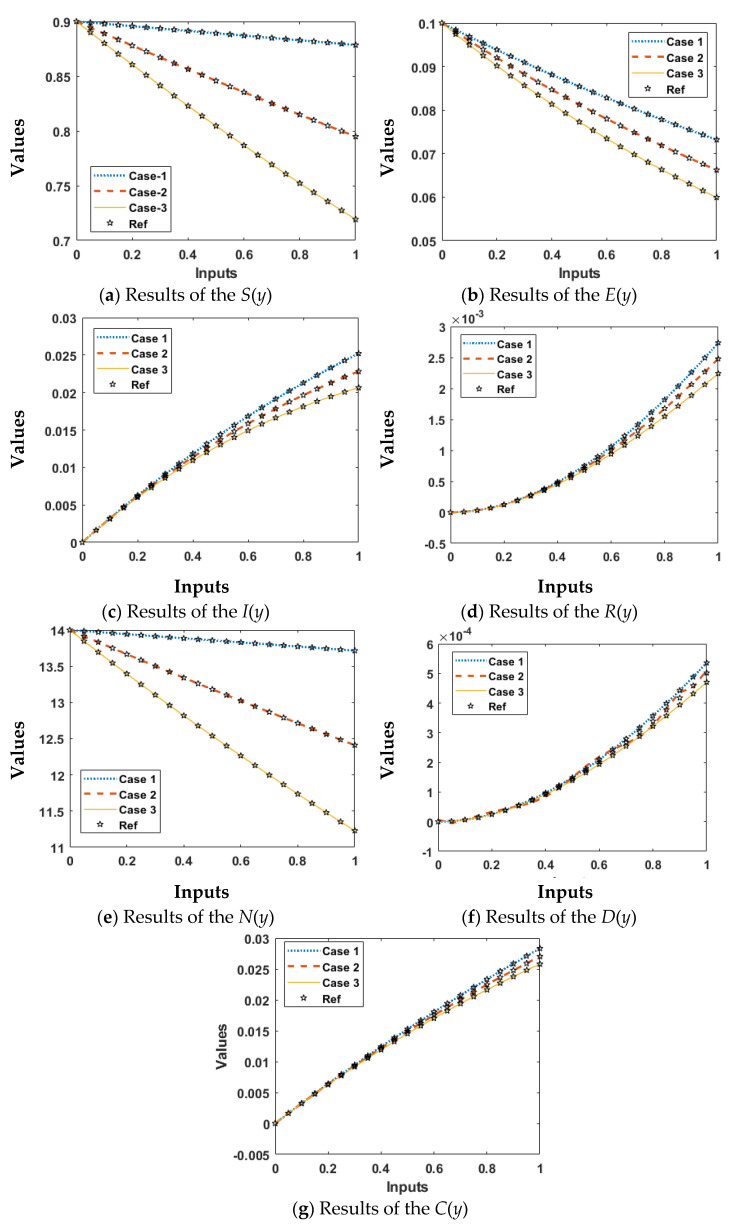
Result comparisons using the ANNs-LMB to solve the nonlinear COVID-19 spreading model.

**Figure 10:**
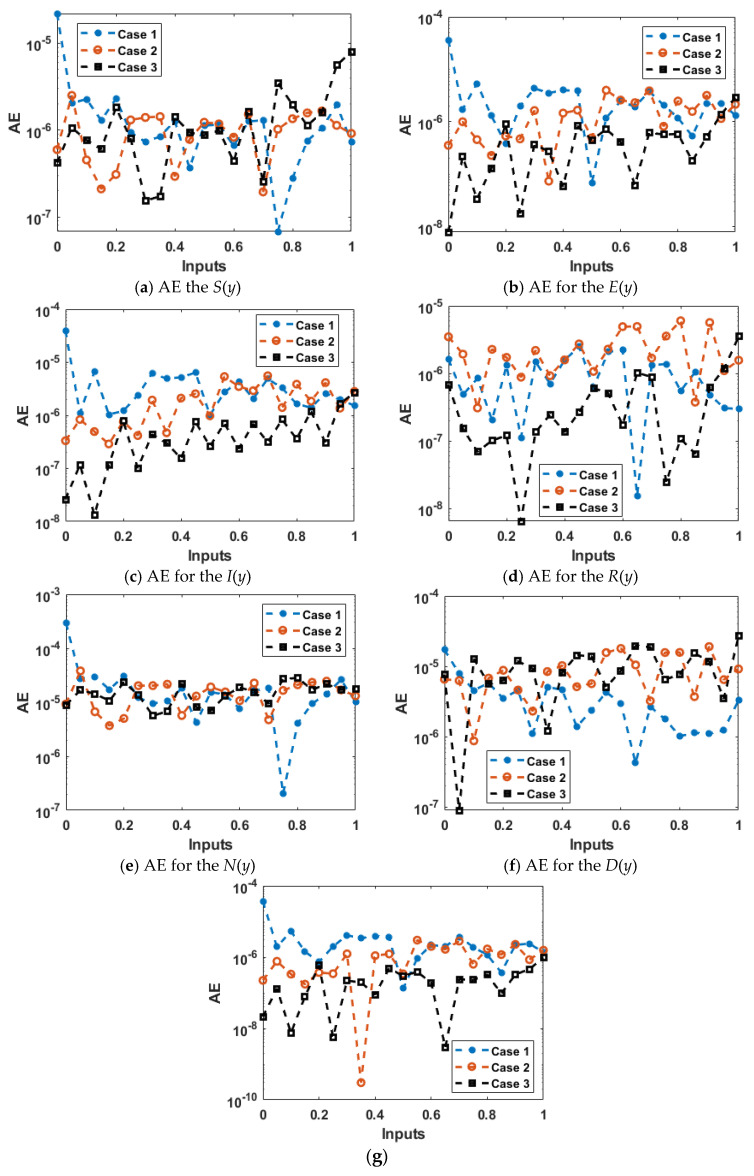
AE using the ANNs-LMB to solve the nonlinear COVID-19 spreading model.

The comparative soundings are demonstrated in Figure 9 and Figure 10 to solve each variant of the nonlinear coronavirus spreading model. The outcomes of the “*S*”, “*E*”, “*I*”, “*R*”, “*N*”, “*D*”, and “*C*” based on the nonlinear coronavirus spreading model using the ANNs-LMB are drawn in Figure 9a–g. The exact (reference and obtained) matching of the results indicates the precision and exactness of the ANNs-LMB to solve each variant of the nonlinear coronavirus spreading model. The AE performances are drawn in Figure 10 to solve each variant of the nonlinear coronavirus spreading model. The AE of “*S*”, “*E*”, “*I*”, “*R*”, “*N*”, “*D*”, and “*C*” based on the based on the nonlinear coronavirus spreading model using the ANNs-LMB are drawn in Figure 10a–g. Figure 10a shows the AE for class “*S*” lie around 10^−5^ to 10^−6^, 10^−5^ to 10^−7^, and 10^−6^ to 10^−7^ for Cases 1–3. Figure 10b indicates the AE for the category “*E*” lies around 10^−5^ to 10^−7^, 10^−6^ to 10^−7^, and 10^−6^ to 10^−8^ for the categories 1–3. It is observed in Figure 10c that the AE for the category “*I*” lie around 10^−5^ to 10^−6^, 10^−6^ to 10^−7^, and 10^−6^ to 10^−8^ for Cases 1–3. Figure 10d provides the results of the AE for the category “*R*” found around 10^−6^ to 10^−8^, 10^−5^ to 10^−7^, and 10^−6^ to 10^−8^ for Cases 1–3. Figure 10e shows the AE values of the dynamics of *N*(*y*) found around 10^−4^ to 10^−6^ for each case of the nonlinear model. In Figure 10f, it is observed the AE for class *“D”* lie around 10^−5^ to 10^−7^ for Case 1, whiles for Cases 2 and 3, these values found around 10^−5^ to 10^−6^. In Figure 10g, it is observed the AE for class *“C”* lie around 10^−4^ to 10^−6^, 10^−6^ to 10^−10^, and 10^−6^ to 10^−8^ for Cases 1–3. These exactly match the results to perform the exactness and perfection of the ANNs-LMB to solve each variant of the nonlinear coronavirus spreading model.

## 4. Conclusions

The models based on the artificial neural networks are presented, together with the training of Levenberg-Marquardt backpropagation, to solve the nonlinear coronavirus spreading model. The numerical scheme based on the ANNs-LMB is implemented for three different procedures of training, authentication, testing, and sample data. These data proportions are provided to solve three variants of the nonlinear coronavirus spreading model, selected as 80%, 10%, and 10% for training, validation, and testing, respectively. The numerical measures have been achieved using the ANNs-LMB, along with the comparative investigations through the reference dataset. The numerical accomplished results from the ANNs-LMB are implemented to reduce the M.S.E. In order to find the exactness, effectiveness, reliability, and capability of the ANNs-LMB scheme, the numerical bases are capable using the proportional actions via M.S.E, correlation, EHs, and regression. The gradient performances using the step size are attained for each variant of the nonlinear coronavirus spreading model. Additionally, the precision and reliability of ANNs-LMB is observed using sufficient large illustrations for numerical and graphical forms through the convergence plots, M.S.E collections, regression dynamics, and EHs.

The designed neuro-evolution based computing strategy is a good alternative to exploit the state of the art on studies related to COVID-19 and dengue modeling and control [39,40,41,42,43].

## Figures and Tables

**Table 1:** ANNs-LMB to solve nonlinear the COVID-19 spreading model.

Case	M.S.E			Gradient	Performance	Epoch	Mu	Time
	Training	Testing	Validation					
**1**	1.37 × 10^−9^	8.74 × 10^−11^	4.71 × 10^−10^	9.84 × 10^−8^	1.37 × 10^−9^	67	1 × 10^−10^	03
**2**	9.62 × 10^−11^	1.85 × 10^−11^	1.33 × 10^−11^	9.99 × 10^−8^	9.62 × 10^−11^	195	1 × 10^−9^	04
**3**	9.93 × 10^−11^	1.18 × 10^−11^	5.71 × 10^−12^	9.92 × 10^−8^	9.93 × 10^−11^	217	1 × 10^−9^	05

## Data Availability

Not applicable.
